# A Single Oscillator-Excited Piezoelectric Actuator with Internal Contact Teeth

**DOI:** 10.3390/mi15010047

**Published:** 2023-12-26

**Authors:** Die Fang, Zhiyi Wen, Zhixin Geng, Xiaopin Hu, Leon Kaswango, Jia Cao, Xiaoniu Li, Dawei Wu

**Affiliations:** State Key Laboratory of Mechanics and Control for Aerospace Structures, Nanjing University of Aeronautics and Astronautics, No. 29 Yudao Street, Nanjing 210016, China; fangdie@nuaa.edu.cn (D.F.);

**Keywords:** helicopter tail rotor drive, piezoelectric actuator, structural and functional integration, drive teeth, single-phase signal

## Abstract

The tail rotor of a helicopter, a crucial component, traditionally relies on a complex drive mode involving reducers and transmission gears. This conventional setup, with its lengthy transmission chain and numerous components, hinders miniaturization efforts. In response to this challenge, our paper presents a novel piezoelectric drive approach. Our objective was to suggest an innovative design capable of minimizing the components involved in the tail rotor drive. This design can be adjusted in size according to specific requirements and is effective up to a specified speed. Moreover, it facilitates the process of miniaturization and integration. The piezoelectric actuator’s stator comprises an ultrasonic amplitude transformer, a ring, and three drive teeth. Utilizing the rod-like structure of the tail brace, the actuator is simplified by adhering ceramic sheets to it. The rotary piezoelectric actuator combines the first longitudinal mode of a rod with torus bending modes. The drive teeth then amplify the ring’s displacement, facilitating rotor rotation. The resonant frequency and modal shape of the actuator were determined using the finite element method. Furthermore, an investigation was conducted to analyze the influence of the drive teeth positioning on the motion trajectory at the contact point. Theoretically, we infer that the declination angle of the drive tooth is a crucial parameter for achieving high speeds. To test our idea, we built three prototype stators with different drive tooth declination angles. Our actuator stands out for its cost-effectiveness, structural simplicity, compatibility with harmonic signals, and ease of miniaturization. It can be considered for the drive of the tail rotor of a microhelicopter.

## 1. Introduction

Helicopters stand out due to their compact size, ability to operate at low speeds, and maneuverability at lower altitudes [1,2]. This compactness allows them to access areas beyond human reach, making tasks like searching for signs of life in intricate environments more feasible [3,4]. The tail rotor of these helicopters serves a dual purpose: it stabilizes flight and adjusts torque balance vertically within the fuselage, thereby enabling changes in flight direction and state and facilitating multi-attitude flying [5]. Nonetheless, the conventional helicopter tail drive system conveys power from the main rotor to the tail using mechanisms such as a belt drive and gear drive. This transmission process involves multiple reducers and transmission shafts, leading to drawbacks, such as a lengthy transmission chain and a high number of mechanical components [6]. Helicopter miniaturization is an emerging trend, necessitating novel forms of propulsion to align with this shift in scale.

Unlike their larger counterparts, unmanned microhelicopters must integrate all components into a smaller space, with stringent weight and power consumption constraints. An analysis comparing the components (electronics, motors, batteries, and airframe) of two differently sized drones [7,8] revealed that miniaturization increases demands on batteries and actuators while still maintaining performance [9]. Therefore, a new type of drive is needed. In the article [10], the author proposes that in the process of further miniaturization of UAVs, the connection of electrical devices on a load-bearing unit will be a promising solution.

A piezoelectric actuator is characterized as a resonant actuator that harnesses the inverse piezoelectric effect within a piezoelectric element. This approach involves exciting the natural vibration of an elastomer and subsequently producing actuation output through frictional coupling [11,12]. Known for their high torque density [13], precise positioning accuracy [14], rapid response, and immunity to electromagnetic interference, these actuators are particularly promising for microrobots [15,16]. The operation of the tail rotor demands a specific rotational speed. Numerous researchers have made noteworthy contributions, highlighting the notable speeds achievable with piezoelectric motors. Chu et al. notably designed a micropiezoelectric actuator employing vibration mode B11 [17]. By employing a piezoelectric disc to induce wobble modes and a metal cylinder stator to augment transverse displacement, they facilitated the rotation of a metal rod rotor. Impressively, this design attained speeds of up to 10,071 rpm at 100 V AC. Borodinas et al. introduced a compact symmetrical coplanar trimorph piezoelectric actuator expressly tailored for high-speed rotary motors [18]. By optimizing the length of the cylinder, they observed a substantial 6.85-fold increase in the amplitude of the contact point oscillation. Through the segmentation of the electrodes of the piezoelectric layers into four equal sections and the application of phase-shifted electric signals, they successfully achieved a rotational speed of 3850 rpm at 80 V. Mashimo engineered an ultrasonic micromotor with a remarkably small volume of only 1 mm^3^, employing a three-wave mode and achieving a peak speed of approximately 2500 rpm [19]. Wang et al. presented a groundbreaking millimeter-sized rotation-type ultrasonic motor featuring a stator with a diameter of 22 mm and a weight of 3.5 g. They accomplished speeds of up to 5520 rpm using a driving voltage of 350 Vp-p [20].

Piezoelectric actuators find application in the design of structurally and functionally integrated systems, leading to a simplification of components and a reduction in overall weight. Wang utilized the first-order torsional vibration and second-order bending vibration of the rod to excite the bending vibration of the ring, facilitating rotor rotation [21]. Simultaneously, the rod-like structure serves as a robotic arm capable of withstanding force loads. Geng introduced an innovative stator-rotor integrated piezoelectric actuator, employing the piezoelectric vibrator as both the driving vibration source and the rotor, thereby streamlining the overall structure of the rotary actuator [22]. Through the utilization of in-plane longitudinal and out-of-plane bending vibrations, the actuator achieves high-precision rotation along the diameter of a circular ring while also providing a hollow structure for optical fibers and wires. Furthermore, piezoelectric actuators have been explored for modifying wing shapes [23,24]. Innovative applications include the use at Harvard University’s Microrobotics Laboratory for tail control of microgliders [25]. The distal end of the actuator is connected to the control surface hinge through a slider crank to form a four-bar mechanism. Thus, the small displacement of the actuator is amplified into a larger rotation at the base of the control surface. In summary, piezoelectric drive technology is expected to be applied to the power source of microhelicopters, especially the tail rotor, which requires a lower speed than the main propeller.

To address these challenges in miniaturizing single-rotor helicopter tail rotors, a piezoelectric drive method is proposed. This involves attaching a ceramic plate to the helicopter’s tail brace, leveraging its unique structure to induce specific vibrational modes and drive the rotor. This approach, by integrating seamlessly with the main system, simplifies the servo drive mechanism significantly. We provide a detailed explanation of the actuator’s operational mechanics and conduct both numerical simulations and optimization exercises. These procedures enable us to determine the optimal positioning of the contact teeth in relation to the ring. After fabricating a prototype, we conducted a series of experimental investigations. A comparison between the experimental results and numerical analyses revealed a consistent alignment. Additionally, we performed performance tests and subsequent analyses after equipping the actuator with a propeller.

## 2. The Design and Operating Principle of the Piezoelectric Actuator

**Figure 1:**
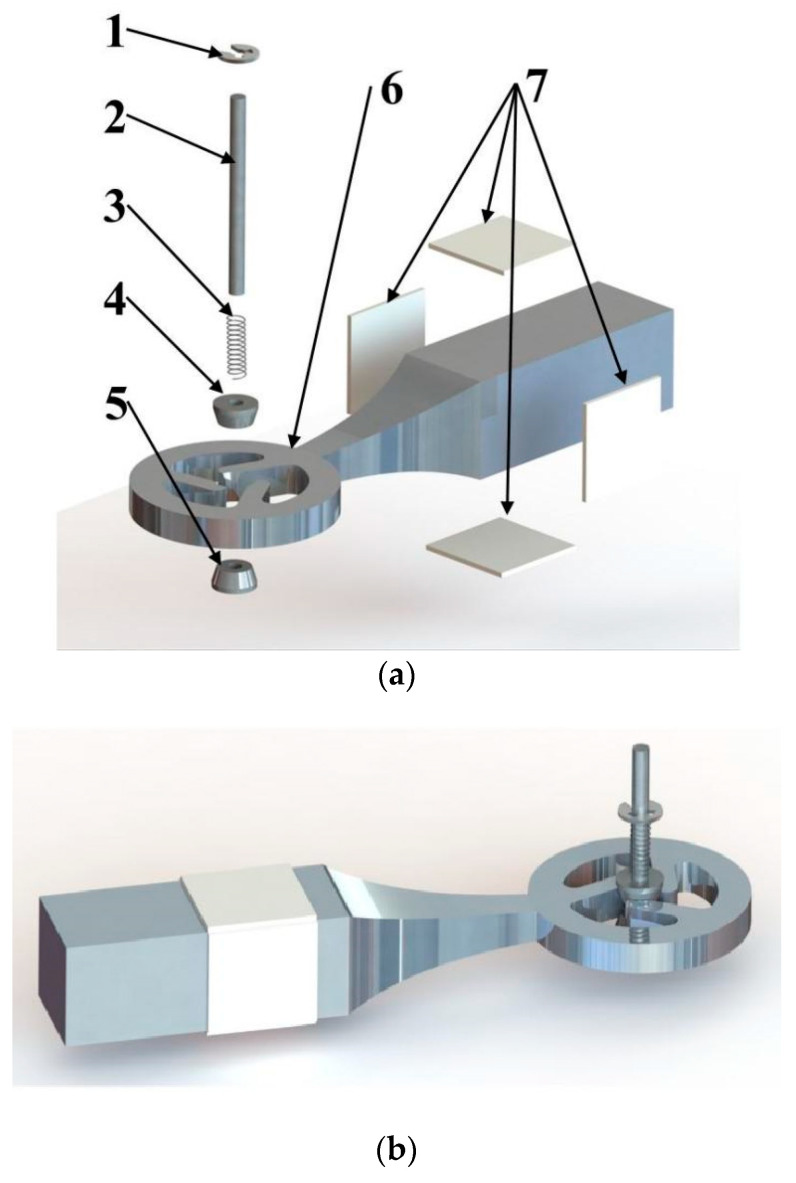
Piezoelectric actuator design: (**a**) Exploded view: 1—circlip, 2—rotation shaft, 3—spring, 4—upper rotor, 5—lower rotor, 6—aluminum alloy rod, 7—ceramic sheets. (**b**) Assembled view of the actuator.

Figure 1 illustrates the design of a single oscillator-excited piezoelectric actuator, which includes several components: a circlip positioned adjacent to a rotating shaft featuring a laser-engraved scale on its side (this scale aids in precise circlip positioning to determine the spring’s length for preload control), a rotating shaft, a spring, an upper rotor (able to slide freely along the shaft), a lower rotor (securely fixed to the shaft), and an aluminum alloy rod with a ceramic sheet.

**Figure 2:**
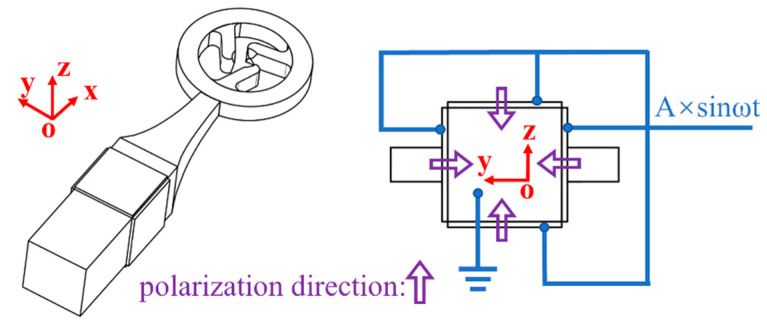
Excitation scheme of actuator.

The stator is composed of an amplitude rod paired with four evenly distributed lateral piezoelectric ceramic sheets. The narrower end of the amplitude rod is linked to the exterior of the ring. Both the amplitude rod and the ring are crafted from a single, unbroken piece of metal, ensuring continuity and integrity. The unique design of the exponential amplitude rod concentrates vibration energy, thereby enhancing the amplitude and vibration velocity of the drive tooth’s surface particles. This concentration significantly boosts the actuator’s mechanical output capabilities. The piezoelectric ceramic sheets are polarized in their thickness direction, as shown in Figure 2. Additionally, the drive tooth’s tip is designed with a symmetrical cone, promoting efficient friction with the rotor. To reduce stress concentration, the base of the drive tooth is chamfered.

The piezoelectric actuator introduced in this study employs a unique integration of the longitudinal vibration of a piezoelectric metal rod with the radial bending of a ring. When subjected to AC voltage excitation, the telescopic vibration of the piezoelectric ceramic sheet initiates longitudinal vibrations within the amplitude rod. By coupling the smaller end of the rod to the ring, these vibrations can be converted into radial bending vibrations, especially when the characteristic frequency of the rod’s longitudinal vibration aligns with the ring’s radial bending mode, denoted as B (0,3). Furthermore, the base of the drive tooth is strategically positioned near the ring’s maximum vibration point, or its “belly”. With the drive tooth oriented at a specific angle relative to the ring’s radial direction, the radial displacement of the ring is transformed into tangential motion, driving the rotation of the rotor.

**Figure 3:**
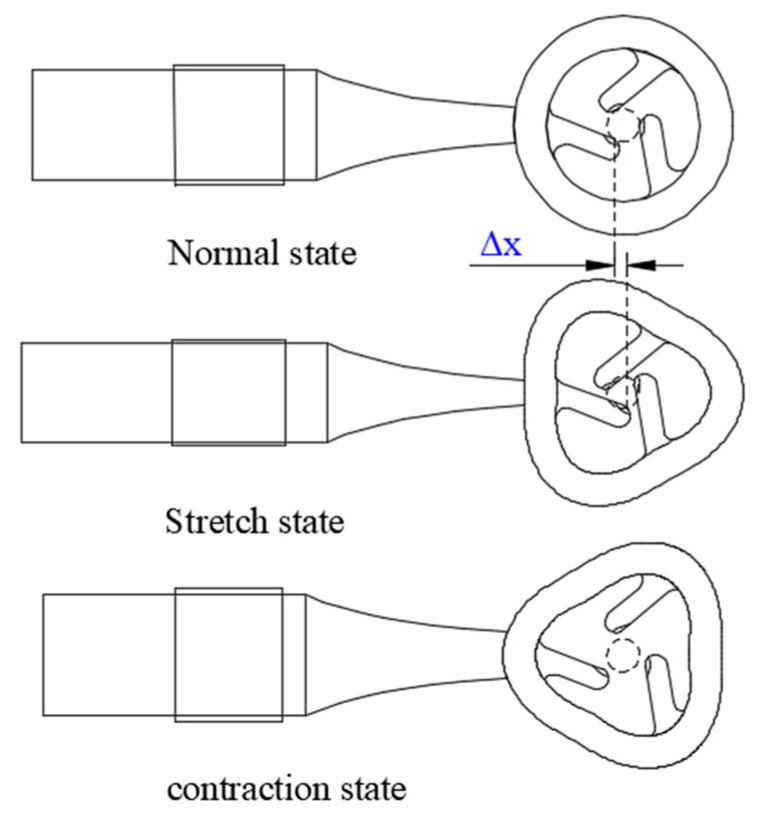
Actuating principle of the actuator.

The actuator’s driving principle primarily relies on a mechanism similar to the “woodpecker” movement of the contact teeth, as illustrated in Figure 3. While the rotor spins at high speeds, it remains mostly separated from the stator. During the stretching phase of the amplitude rod, the drive teeth advance by a distance of ∆*x*, initiating the rotor’s rotation. Conversely, as the amplitude rod contracts, the drive teeth move away from the rotor. The rotor, relying on its inertia, continues its rotational motion.

**Figure 4:**
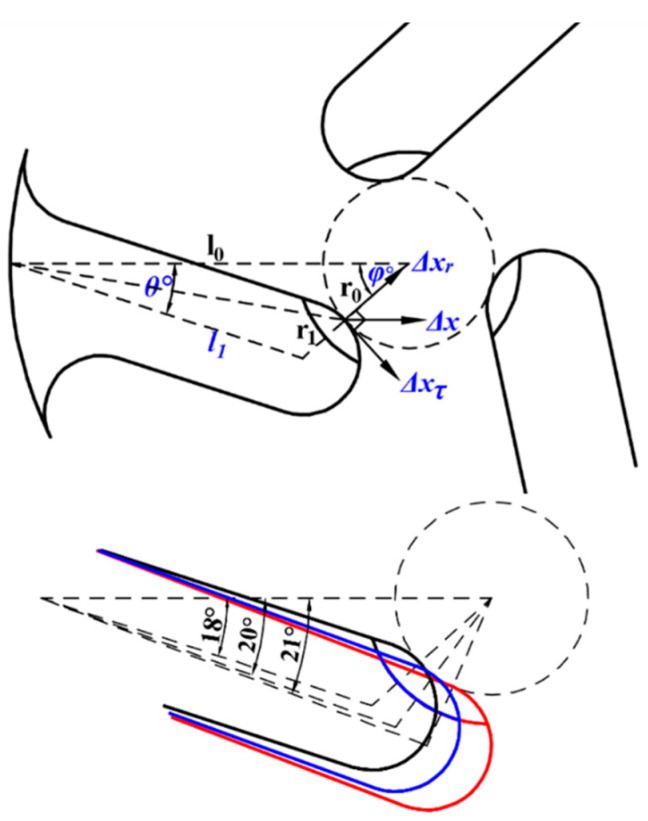
Stator and rotor contact.

In the design, three parameters remain constant: the width r_1_ of the teeth, the inner diameter of the ring *l*_0_, and the radius *r*_0_ of the circle inscribed within the three teeth. As depicted in Figure 4, the angle *θ* defines the tangent point at the tooth tip in relation to the inscribed circle. By applying the sine rule for triangles, we can establish the following relationship:

(1) $$\frac{l_{1}}{\sin\varphi }=\frac{r_{0}+r_{1}}{\sin\theta }$$

The angle *θ* is constrained within specific limits. If *θ* is too small, the force in the tangential direction becomes insufficient for the rotor to initiate rotation. Conversely, if *θ* is excessively large, the rotor and drive teeth fail to make contact, preventing the transmission of force. When the drive teeth align tangentially with the rotor, a maximum angle of 21° is reached, as illustrated in Figure 4 (the red line). In essence, once the value of *θ* is established, both the values of *φ* and *l*_1_ are concurrently determined.

The displacement’s tangential component, denoted as ∆*x_τ_*, in relation to ∆*x*, is given by:

(2) $$\Delta x_{\tau }=\Delta x\times \sin\varphi =\Delta x\times \sin(\theta +\arcsin\frac{l_{0}\times \sin\theta }{r_{0}+r_{1}})$$

It is ∆*x_τ_* that propels the rotor to turn. As *θ* increases, ∆*x_τ_* likewise increases, implying that, in theory, the rotor’s speed should also rise with an increasing *θ*.

## 3. Numerical Modeling and Results

**Figure 5:**
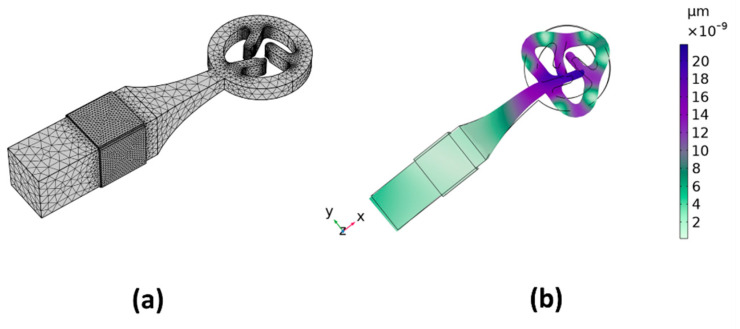
(**a**) Finite element method (FEM) mesh of the stator; (**b**) mode shape of the stator.

Using numerical simulations, this study investigates the influence of varying geometries and parameters on structural properties. A primary objective of these simulations is to determine the characteristic frequency and mode shape of the stator. To achieve this, the stator’s numerical representation was developed using COMSOL Multiphysics 6.0 FEM software. Figure 5a displays the finite element model of the stator, consisting of an aluminum alloy block and four piezoelectric ceramic sheets. All four piezoelectric ceramic sheets have a uniform thickness of 0.5 mm, sourced from Shengnuo Company in Zhongshan, China. It is important to note that, for simplicity, the adhesive layers were omitted from this model. The meshing of the stator was performed using a three-dimensional standard tetrahedral finite element approach. After multiple iterations to ensure model accuracy and fidelity, the finalized mesh included 59,556 domain elements, 13,612 boundary elements, and 1132 edge elements. Table 1 provides a comprehensive list of material properties for both the aluminum alloys and piezoelectric ceramics.

**Figure 6:**
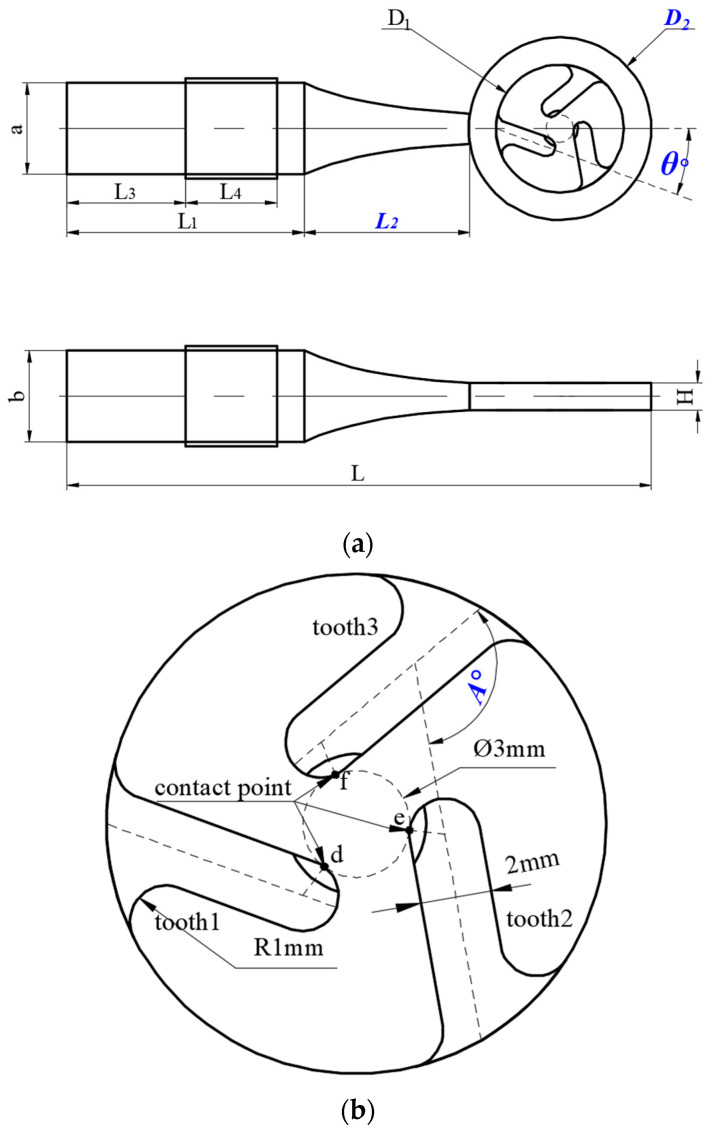
(**a**) Size parameters of the stator; (**b**) local enlargement of the stator.

The primary objective of the initial optimization step is to synchronize the rod’s first-order longitudinal vibration with the ring’s third-order in-plane vibration. To achieve this, certain geometric parameters of the stator require optimization, with all relevant variables depicted in Figure 6a. Within the design constraints, the rotor’s diameter and the drive teeth’s width remain constant. The declination angle and the drive teeth’s lengths are interdependent, with the initial declination angle, *θ*, set at 20°. Among all the parameters, *L*_2_ has the greatest influence on the rod’s first-order longitudinal vibration, while *D*_2_ is crucial for the ring’s bending vibration. Through a comprehensive parametric sweep, we identified an optimal modal shape at 67.43 kHz, as illustrated in Figure 5b.

**Figure 7:**
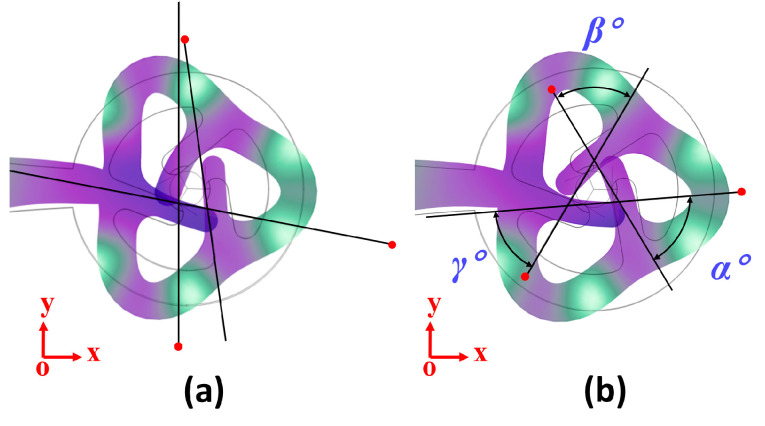
(**a**) Trajectory before optimization; (**b**) optimized trajectory.

After establishing the stator’s working mode, an analysis of the contact trajectory revealed an unbalanced motion trajectory for the drive teeth, as depicted in Figure 7a. The motion of the three drive teeth exhibits inconsistency, leading to a counteractive force output and influencing the ultimate speed. Notably, we observed that the deformation of tooth 3 differed significantly from that of the other two teeth. Consequently, the objective of the second optimization was to modify the relative position of tooth 3. The subsequent optimization step aims to determine the optimal relative positions of the three drive teeth. To facilitate this optimization, we employed the frequency-domain-to-time domain analysis method. This method was utilized to compute the trajectories of the three contact points, which closely resemble three straight lines. The points where two of these lines intersect form intersection points, subsequently creating a triangle when all three points are connected. The respective angles of this triangle are denoted as *α*, *β*, and *γ*. The definition for *δ* is as follows:

(3) δ=180−(|α−60|+|β−60|+|γ−60|)

**Figure 8:**
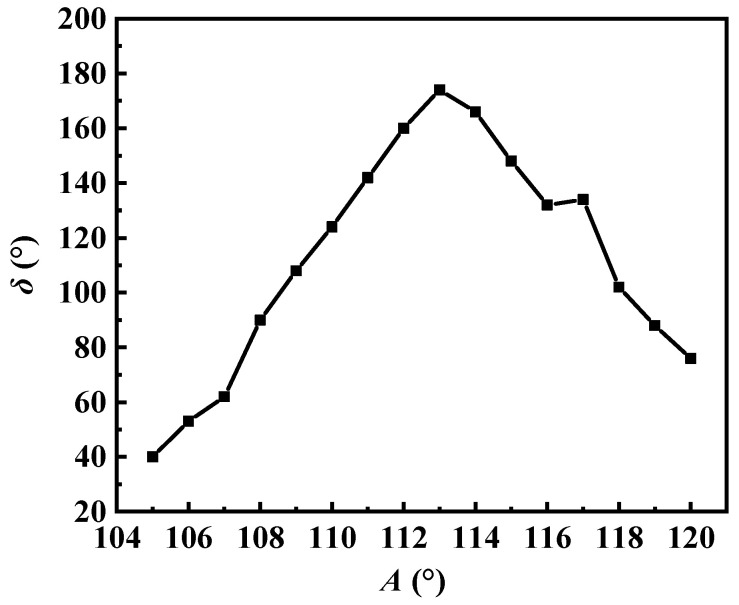
*δ* versus the angle *A*.

As shown in Figure 6b, tooth 2 is derived by rotating tooth 1 counterclockwise by 120°. Similarly, tooth 3 is derived from tooth 2 through a counterclockwise rotation of *A°*. Through simulations, the value of *δ* is determined as *A* varies from 105° to 120°, and the results are depicted in Figure 8. The data clearly indicate that when angle *A* equals 113°, the triangle formed by the three points closely resembles an equilateral triangle. The results of this optimization process are presented in Figure 7b.

**Figure 9:**
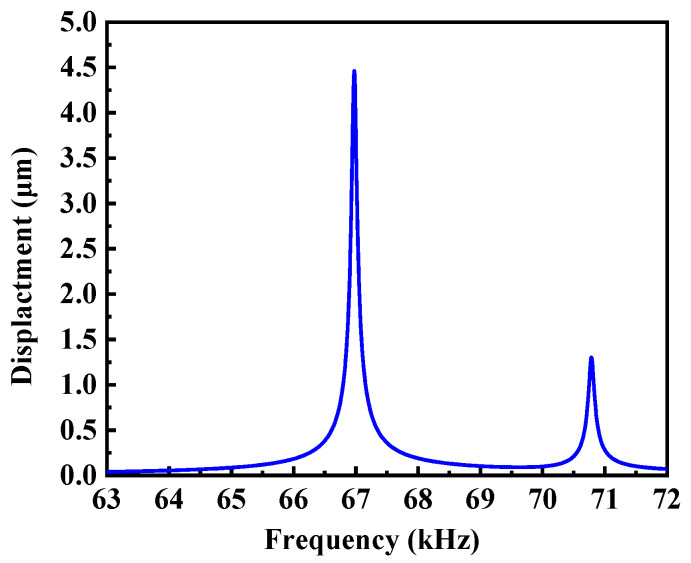
Contacting point oscillation amplitude versus frequency.

**Figure 10:**
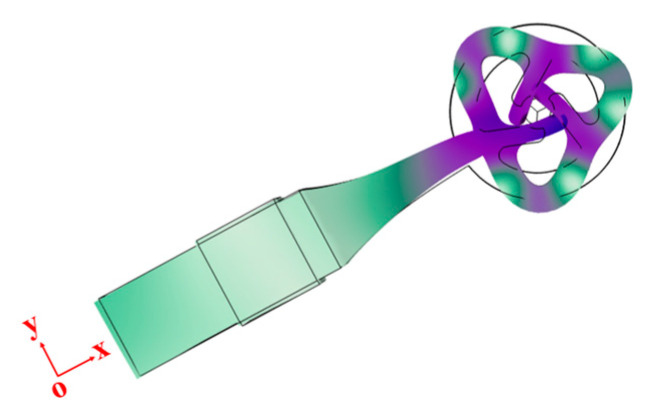
Mode shape of the stator.

By employing harmonic response analysis, we identified the resonant frequency and computed the motion trajectory of the contact point under alternating voltage. In the simulation, the electrodes were subjected to a 40 Vp-p signal. The frequency exploration ranged from 62 kHz to 72 kHz, with a variable step size ranging from 1 Hz to 10 Hz, depending on the specific internal frequency range. The relationship between displacement and frequency can be observed in Figure 9. An optimal resonance frequency of 66.97 kHz emerged, accompanied by its corresponding vibration shape for the actuator, as illustrated in Figure 10. The vibration mode at approximately 71 kHz corresponds to the third-order bending vibration of the rod. This frequency is approximately 4 kHz apart from the intended mode, and it does not interfere with or impact the utilization of the target mode.

**Figure 11:**
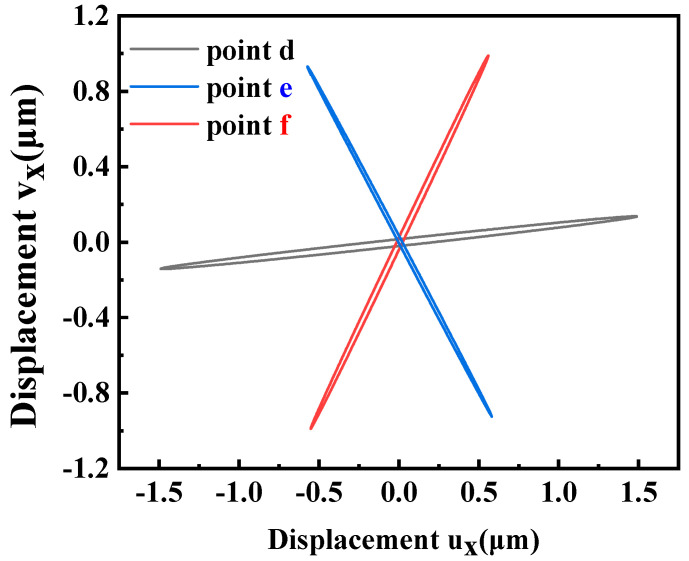
Contact points trajectories in xy plane.

Using transient analysis, we examined the motion trajectory of the driving teeth within the stator ring. Figure 11 illustrates that the trajectories of all three teeth collectively resemble a flattened ellipse, closely approximating a straight line. Furthermore, all three points simultaneously approach and recede from the circle’s center, aligning with the previously outlined driving principle. 

## 4. Experimental Study

**Figure 12:**
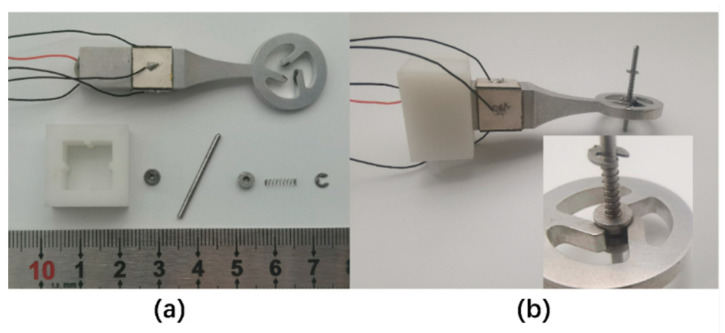
(**a**) Components for the piezoelectric actuator. (**b**) Assembly results.

To validate the simulation optimization results and assess the reliability of piezoelectric actuators, we constructed a prototype. The stator was fabricated following the finalized structural parameters detailed in Table 2, which were derived from the simulation analysis. The drive teeth of the stator were shaped using wire EDM, and to ensure efficient displacement transmission to the rotor, the tips of the teeth were designed with a tapered chamfer. The rotor was crafted from stainless steel. As shown in Figure 12, we assembled a prototype of the piezoelectric actuator. PZT was affixed to the stator using epoxy resin, resulting in a total weight of 14 g for the prototype.

### 4.1. Impedance Analysis Test

**Figure 13:**
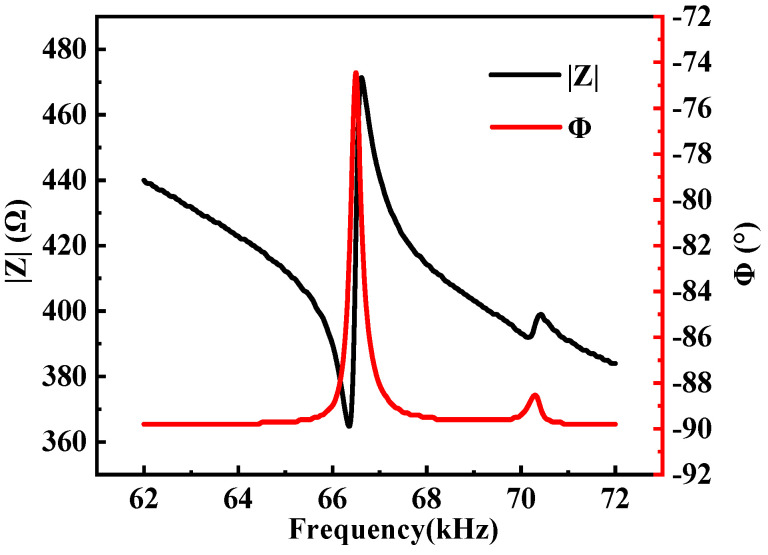
Impedance-frequency characteristics of the stator.

First and foremost, it was crucial to determine the resonant frequency of the stator. The frequency response of the measured impedance indicates the existence of natural frequencies: the impedance of the stator gradually decreases at resonance. We assessed the input impedance characteristic of the prototype using the impedance analyzer (E4990A, Keysight, Guangzhou, China), setting the frequency range between 62 kHz and 72 kHz. This analysis is performed by connecting a piezoelectric sheet and stator to the high and low electrodes of an impedance analyzer, where the voltage amplitude is set to 5 Vp-p. The results are depicted in Figure 13: the black line represents the |Z|-f curve (where |Z| signifies the stator’s impedance), while the red line indicates the Φ-f curve, with Φ representing the phase shift. These findings highlight a characteristic frequency of 66.45 kHz for the prototype. The observed frequency differs from the analog frequency by approximately 0.78%. Such discrepancies mainly result from variations in actual material parameters compared to simulation settings, as well as processing and assembly inaccuracies.

### 4.2. Vibration Mode Test

**Figure 14:**
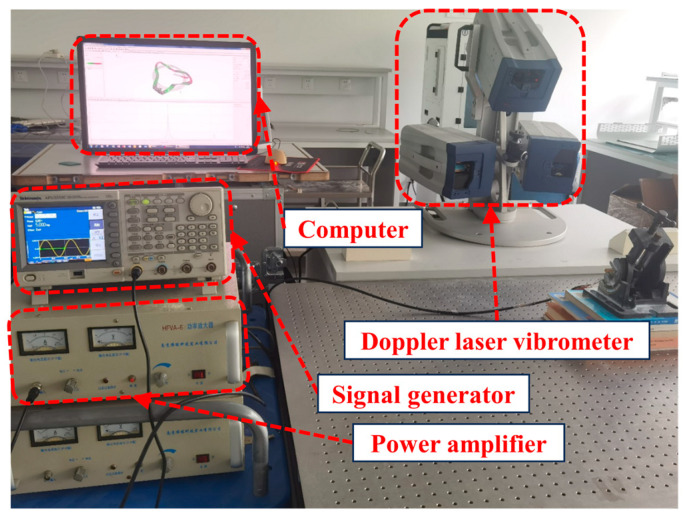
Experimental device for vibration characteristics.

Our next objective was to verify if the stator’s operating mode aligns with the FEM simulation outcomes. For this evaluation, we employed a set of tools: a three-dimensional laser Doppler vibrometer (PSV-500-3-D, POLYTEC Inc., Waldbronn, Germany), a computer, a power amplifier, and a signal generator, as illustrated in Figure 14. Due to structural constraints, we selected the end face of the ring as the testing surface. The four piezoelectric ceramic sheets were subjected to a sinusoidal signal with a voltage of 30 Vp-p, sweeping through frequencies ranging from 60 kHz to 72 kHz.

**Figure 15:**
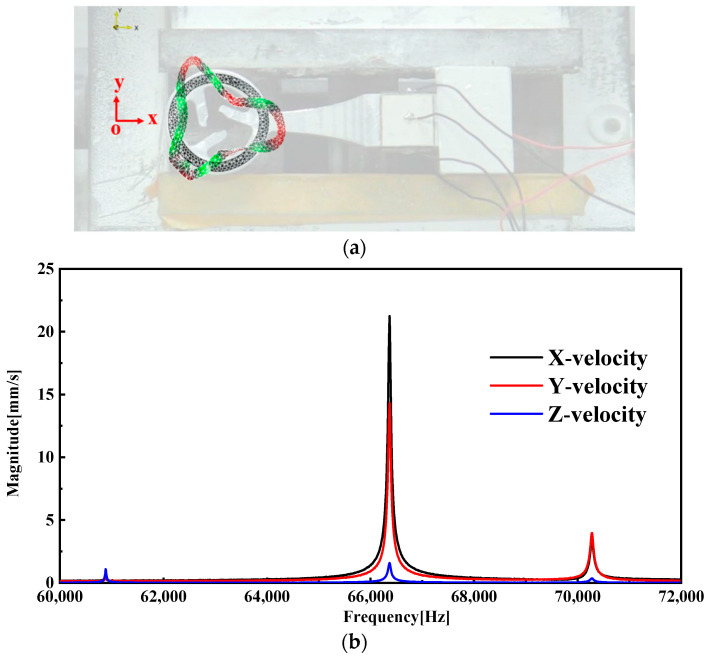
(**a**) Mode shape. (**b**) Velocity–frequency curve.

Figure 15 illustrates both the mode shape diagram and the velocity–frequency characteristic curve. The observations indicate that the B03 vibration mode predominantly influences the vibration shape of the ring. There is minimal displacement in the Z direction, suggesting that the majority of the vibration occurs within the x–y plane. The mode shapes of the end faces of the stator ring can be viewed in the supplementary materials. Importantly, the point of maximum displacement in the ring is located at the base of the drive teeth, aligning with our expectations. The recorded resonant frequency is 66.36 kHz, closely matching our simulation results.

### 4.3. Actuator Performance Test

**Figure 16:**
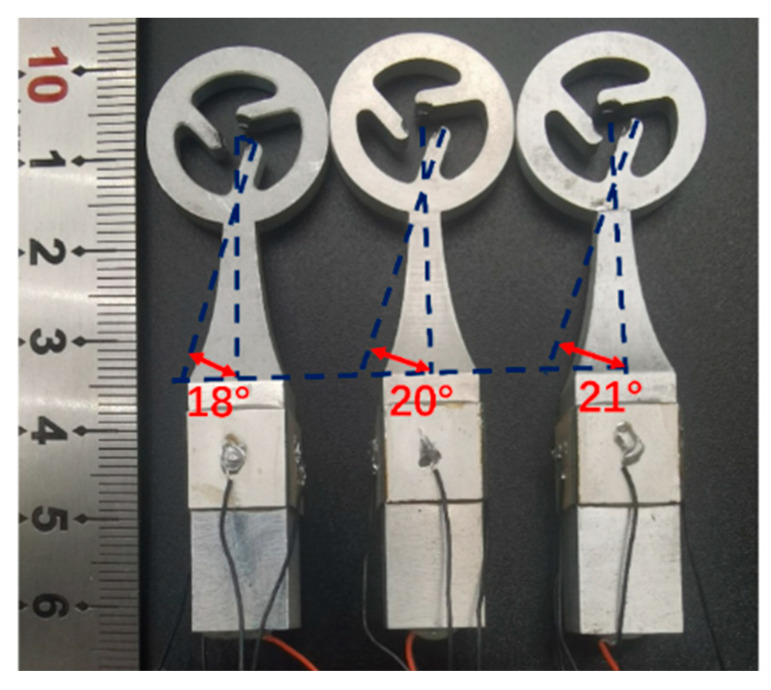
Stators with different declination angles.

To examine the influence of the declination angle of the drive teeth on rotational speed, we constructed three stators with different declination angles: 18°, 20°, and 21°, as depicted in Figure 16.

**Figure 17:**
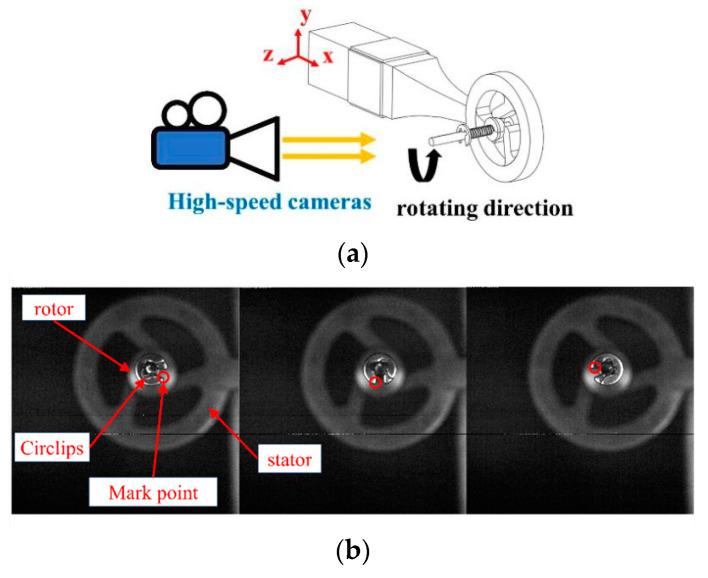
(**a**) Schematic diagram of the speed measuring device. (**b**) The high-speed camera takes pictures of actuator rotation.

**Figure 18:**
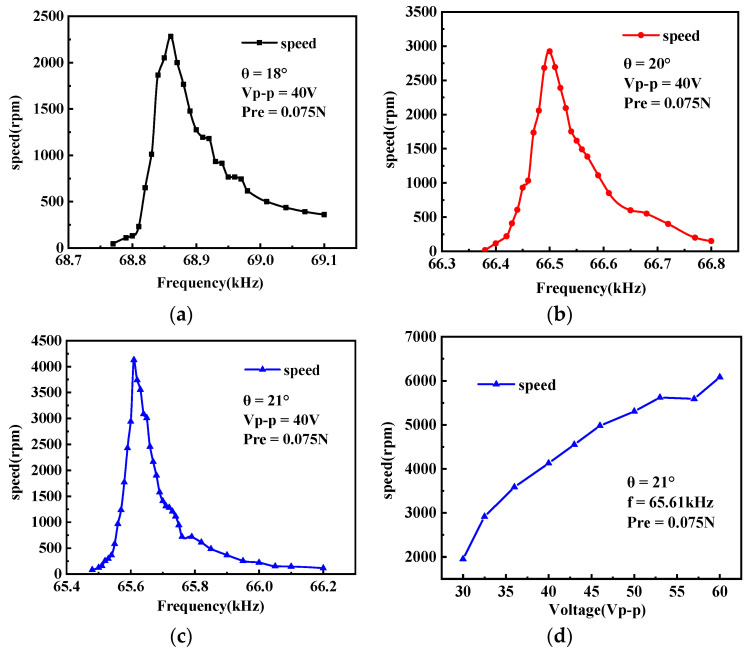
(**a**–**c**) The measured rotational speed versus frequency; (**d**) the measured rotational speed versus voltage.

Subsequently, we evaluated the mechanical output characteristics of the prototype. Figure 17a illustrates a schematic diagram of the device used to measure rotational speed. The rotating actuator is captured using a high-speed camera (DIMAX HS4, PCO, Cologne, Germany) at a rate of 2000 frames per second. The actuator’s rotational speed is determined by calculating the time it takes for the actuator to rotate through a known angle, derived from the number of frames captured during that interval. Figure 17b presents a selection of images captured by the high-speed camera, illustrating the actuator’s rotation process. The amount of prepressure can be adjusted by adjusting the position of the circlip. The stiffness coefficient of the spring is 0.05 N/mm. During the tests, the AC excitation voltage supplied by the piezoelectric ceramic sheet was set at 40 Vp-p, and the prepressure exerted between the stator and rotor was maintained at 0.075 N. Figure 18a–c displays the speed curves for the stator at varying declination angles under no-load conditions. It is evident from the graphs that as the declination angle increases, the rotational speed of the stator also increases.

To explore the relationship between actuator speed and drive voltage, we analyzed the test results and identified that the actuator with a declination angle of 21° exhibited superior performance. Consequently, this actuator was selected for subsequent tests. The experimental setup remained consistent with previous tests, maintaining a preload of 0.075 N and an operating frequency of 65.61 kHz. Figure 18d illustrates the connection between rotational speed and voltage. Remarkably, within a specific range, there is an approximately linear increase in the no-load speed as the voltage rises, suggesting that actuator speed can be controlled by adjusting the input voltage.

**Figure 19:**
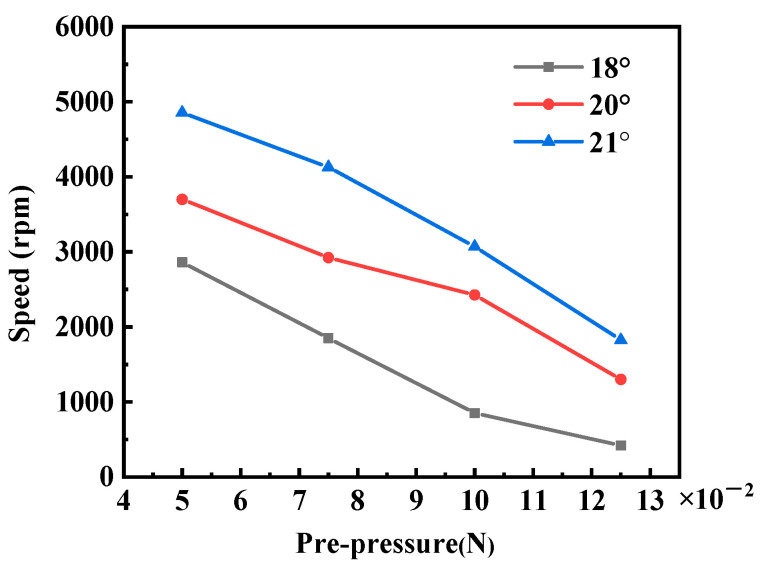
The measured rotational speed versus prepressure.

Furthermore, altering the declination angle of the drive tooth changes the effective contact area between the drive tooth and the rotor. To assess the impact of these variations in contact area on the optimal working prepressure for the stator, we examined the relationship between speed and prepressure for stators with different declination angles. These actuators were tested at their respective optimal operating frequencies with a drive voltage of 40 Vp-p. The depicted results in Figure 19 illustrate that, with a constant angle, the rotational speed decreases as the prepressure increases within a specific range. Likewise, with a constant prepressure, a decrease in the angle within a certain range is associated with a corresponding decrease in rotational speed. This suggests that, within the tested range, the influence of prepressure on actuator speed remains consistent, irrespective of the variations in contact area due to differing declination angles.

### 4.4. Actuator Lift Test

**Figure 20:**
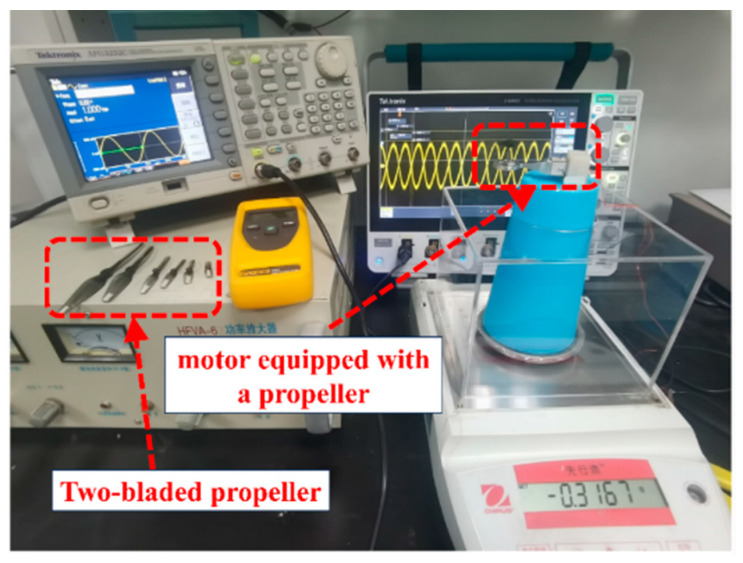
Lift test system.

In the following phase, we evaluated the actuator’s load characteristics. For this assessment, we selected an actuator with a declination angle of 21° to evaluate its lifting capability. We examined both the lift and speed of the actuator equipped with various blades. Figure 20 illustrates the experimental setup for measuring rotor lift, which includes a power amplifier, an oscilloscope, a rotor prototype, and a high-precision electronic scale. The prototype was securely fixed to the base using a hot melt adhesive. This base was then positioned on the measuring platform of the scale, and its reading was zeroed. As the rotor initiates rotation, it generates an upward thrust. Consequently, a negative reading on the scale indicates the lift currently provided by the rotor. To minimize the impact of airflow on the readings, we incorporated a draft shield into the electronic scale. It is worth noting that we used a double-blade propeller for the tests, as research indicates its superior efficiency in micro-aircraft applications. The movement of the rotor with the propellers attached can be viewed in the supplementary materials.

**Figure 21:**
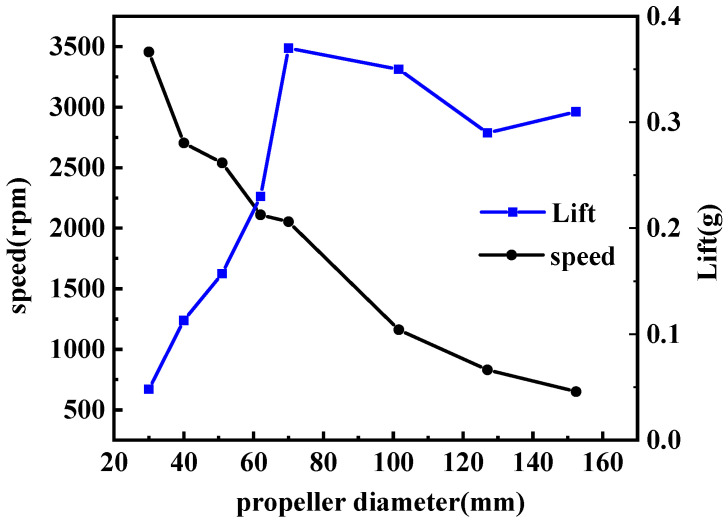
Speed and lift versus propeller diameter.

Throughout the experiment, the actuator operated at its optimal frequency with a voltage set at 40 Vp-p and a prepressure of 0.075 N. Figure 21 illustrates the test results. As the blade diameter increases, there is a noticeable decrease in rotational speed. Simultaneously, the lift experiences a rapid increase followed by a gradual decline. This rise in the curve is attributed to the improved lift provided by larger paddles. However, the subsequent decline suggests that a reduction in speed has a more significant impact on lift than the increase in blade area. Based on these findings, the prototype appears best suited for driving a double blade with a 70 mm diameter.

Table 3 presents a comparative analysis between the rotary piezoelectric actuators proposed in this study and other previously documented piezoelectric rotating motors. A notable distinction lies in the driving signal requirements, as our actuator necessitates only a single-phase drive, streamlining control in comparison to the other listed actuators. Furthermore, our actuator features a larger rotor diameter, enhancing shaft stiffness and its capacity to withstand torsional forces. Additionally, it achieves higher speeds at a lower voltage. In summary, the piezoelectric actuator introduced in this study stands out with the advantages of a simplified drive signal and a high rotational speed.

## 5. Conclusions

This actuator achieves remarkable rotational speeds by harnessing the displacement amplification principle of ultrasonic amplitude transformers in conjunction with drive teeth. Both numerical simulations and experimental results validate the actuator’s operational principle. We analyzed the vibrations at the contact points, ensuring unidirectional movement of the rotor. Additionally, we introduced a methodology to optimize the relative positioning of the driving teeth, which was confirmed through numerical modeling. Comprehensive investigations elucidated the relationships among rotational speed, frequency, voltage, and prepressure. The most efficient prototype has a declination angle of 21° and operates at a frequency of 65.61 kHz at 40 Vp-p, achieving a peak no-load speed of 4100 rpm. When assessing blades of varying diameters, the actuator generated a lift of 0.37 g at a speed of 2100 rpm.

Remarkably, the actuator’s structure can serve as the aircraft’s fuselage, offering applications in tail rotors for helicopters. Ceramic sheets are affixed to the tail brace to induce the desired vibration mode, subsequently driving the tail rotor to rotate through friction. This approach is beneficial in minimizing the number of drive components and decreasing the structural weight of the tail rotor drive. It facilitates the integration of structure and function, ultimately enabling miniaturization. Further reductions in overall dimensions can be achieved by employing a more compact stator and PZT. In the future, our research efforts will focus on improving actuator performance through strategies such as refining the stator’s architecture and utilizing lightweight materials like carbon fiber.

## Figures and Tables

**Table 1:** Properties of the materials.

Material Property	Aluminum	PZT-8
Density (kg/m^3^)	2700	7600
Young’s modulus(N/m^2^)	7 × 10^10^	-
Poisson’s coefficient	0.33	-
Relative permittivity	-	$\left[\begin{array}{ccc} 904.4 & 0 & 0\\ 0 & 904.4 & 0\\ 0 & 0 & 561.6 \end{array}\right]$
Elastic compliance coefficient (C/m^2^)	-	$\left[\begin{array}{cccccc} 0 & 0 & 0 & 0 & 10.3 & 0\\ 0 & 0 & 0 & 10.3 & 0 & 0\\ -3.9 & -3.9 & 14.0 & 0 & 0 & 0 \end{array}\right]$
Elastic stiffness coefficient (10^10^ N/m^2^)	-	$\left[\begin{array}{cccccc} 14.7 & 8.1 & 8.1 & 0 & 0 & 0\\ 8.1 & 14.7 & 8.1 & 0 & 0 & 0\\ 8.1 & 8.1 & 13.2 & 0 & 0 & 0\\ 0 & 0 & 0 & 3.1 & 0 & 0\\ 0 & 0 & 0 & 0 & 3.1 & 0\\ 0 & 0 & 0 & 0 & 0 & 3.3 \end{array}\right]$

**Table 2:** Main dimension parameters of the stator.

Parameters	a	b	L	L_1_	*L*_2_	L_3_	L_4_	D_1_	*D*_2_	H	*θ*
Values(mm)	10	10	64	26	18	14	10	3	1	3	20°

**Table 3:** Comparison of other piezoelectric rotary motors.

Author	Driving Signal	Driving Voltage	Speed	Rotor Diameter
This work	Single phase	40 Vp-p	4100 rpm	3 mm
Chu et al. [17]	Two phases	100 Vp-p	10,071 rpm	1 mm
Borodinas et al. [18]	Four phases	80 Vp-p	3850 rpm	0.32 mm
Mashimo [19]	Two phases	98 Vp-p	2500 rpm	0.7 mm
Wang et al. [20]	Four phases	350 Vp-p	5520 rpm	2.75 mm

## Data Availability

The data that support the findings of this study are available from the corresponding author upon reasonable request.

## Supplementary Materials

The following supporting information can be downloaded at: https://www.mdpi.com/article/10.3390/mi15010047/s1, Video S1: Mode shape of the end face of the stator ring; Video S2: The rotor in operation with its blades.
